# Thymoma with recurrent opportunistic infections—a case report

**DOI:** 10.1093/omcr/omae102

**Published:** 2024-09-07

**Authors:** Sri Lasya Karjala, Satya Prasad Namala, Phani Krishna Machiraju, Prabu Pandurangan

**Affiliations:** Department of General Medicine, Apollo Hospitals, Chennai 600 006, Tamilnadu, India; Department of Hematology, Apollo Hospitals, Chennai 600 006, Tamilnadu, India; Department of Hematology, Apollo Hospitals, Chennai 600 006, Tamilnadu, India; Department of Hematology, Apollo Hospitals, Chennai 600 006, Tamilnadu, India

**Keywords:** thymoma, opportunistic infections, good’s syndrome, hypogammaglobulinemia

## Abstract

Good’s syndrome (GS) is a rare adult-onset thymoma associated with acquired combined B-cell and T-cell immunodeficiency. It has similarities with Common variable immunodeficiency (CVID) in terms of hypogammaglobulinemia and significant risk of invasive bacterial and opportunistic infections. We still have a long way to go in understanding the pathogenesis of Good’s syndrome. Here, we describe a case of a middle-aged female with thymoma and recurrent opportunistic infections. Clinico-laboratory evaluation led to a diagnosis of GS and she showed good response to intravenous immunoglobulin. Clinicians should be aware that thymoma can precede the onset of immunodeficiency.

## Introduction

Good’s syndrome (GS) is a rare adult-onset thymoma associated with acquired combined B-cell and T-cell immunodeficiency. Since it was first described by Robert A. Good in 1954 [1], not more than 200 patients were reported in the literature [2]. Around 0.2–6% of thymomas are associated with Good syndrome. A variety of immunodeficiencies are seen in patients with GS like low to total absence of peripheral B cells with hypogammaglobulinemia, variably altered T-cell populations, CD4/CD8 ratio reversal, CD4 lymphopenia, and variable proliferative response to mitogen.

The association between thymoma and immunodeficiency is not very clear. Many different theories were proposed on the pathogenesis of Good’s syndrome but so far none has gained acceptance, making this syndrome still a mystery. The exact role of thymoma in Good syndrome development remains unclear; it likely disrupts the balance between host defenses and self-tolerance [3]. Autoimmune manifestations like pure red cell aplasia, myasthenia gravis and lichen planus are a few conditions that can be associated with this condition.

Studies have showed that GS is associated with an increased susceptibility to infections. The cytokines released by bone marrow stromal cells impair both thymic and B cell precursor growth and differentiation; thymic T cells exert inhibitory effect on B cell mediated immunoglobulin production [2]. Good’s syndrome remains a challenge for treating physicians due to its wide range of clinical and immunological heterogeneity. There are currently no established treatment protocols. We describe a case of a 53-year-old female with Type AB thymoma and history of recurrent opportunistic infections following thymectomy.

## Case details

This 53-year-old Indian Woman with no significant past medical history, presented elsewhere with complaints of persistent dry cough and fever for one month. Computed tomography scan chest revealed mediastinal mass, which was excised and histopathological examination revealed a type AB thymoma.

She developed a non-healing oral lesion four months post surgery. She was diagnosed to have lichen planus based on the histopathological examination, which resolved on treatment with cyclosporine. Over the next three months, she had persistent fever, loss of appetite, weight loss and dysphagia. Chest X-ray revealed bilateral lung consolidation and broncho-alveolar lavage samples were sent for microbiological examination and cytomegalovirus (CMV) polymerase chain reaction (PCR) was positive. Oesophago-gastro-duodenoscopy revealed esophageal candidiasis. She was treated with valacyclovir and fluconazole following which she improved clinically. Two months later, she presented to our hospital with persistent diarrhea, fever and weight loss.

Baseline investigations revealed mild leukocytosis, eosinopenia, low protein levels and low globulin levels. Human immunodeficiency virus serology was negative. Enteropathogenic Escherichia. Coli (EPEC), *Giardia lamblia* and *cryptosporidium parvum* were detected in her stool sample by multiplex PCR technique and stool cultures. She was treated with azithromycin, tinidazole and nitazonaxide, she recovered well.

In view of recurrent infections in the background of thymoma her serum immunoglobulin levels were checked, and they were significantly low (Table 1). Flow cytometry of peripheral blood for B and T cell marker revealed a complete absence of B cells while CD4, CD8 count and ratio of CD4/CD8 cells were normal (Table 2). In view of recurrent infections, hypogammaglobulinemia and total absence of B-cells in the background of thymoma she was diagnosed to have GOOD’S SYNDROME.

**Table 1:** Serum Immunoglobulin levels of our patient

**Type of Immunoglobulin**	**Patient levels**	**Normal range**
IgG	178	650–1640 mg/dl
IgA	<26.1	52–468 mg/dl
IgM	<17.5	39–338 mg/dl

**Table 2:** Flow cytometry of the peripheral blood

Lymphocyte subsets	%	Absolute counts/ul	Reference range
Total leucocyte		9391	4000–11 000
Total Lymphocyte	18.20	1709	1836–2977
Total T lymphocyte	81.34	1390	1413–2379
Total B cells	0	0	90–413
NK cells	14.02	240	150–433
CD4+ T cells	39.71	679	815–1769
CD8+ T cells	38.16	652	194–1432

**Figure 1:**
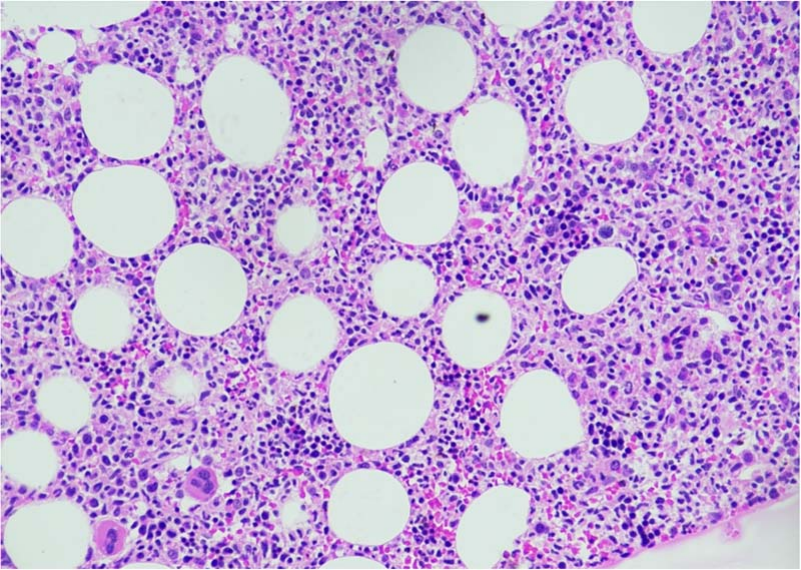
Bone marrow biopsy showing hypercellular marrow with trilineage hematopoiesis.

A bone marrow aspiration and biopsy was done to rule associated hematological conditions like myelodysplastic syndrome, pure red cell aplasia. It was hypercellular with no evidence of dysplasia or atypical cells (Fig. 1) and her reticulocyte count was 4%. She was started on once a month low-dose intravenous immunoglobulin (0.4 g/kg). She showed good response to treatment in the form of weight gain and reduced frequency of opportunistic infections and hospitalizations.

## Discussion

GS is classified as a distinct entity by the World Health Organization and it is mainly diagnosed by the association between thymoma, hypogammaglobulinemia, and decreased or absent peripheral B cells. GS is seen in 3%–6% of patients with thymoma [4]. Most patients with GS present at 40–60 years of age, similar to our patient who presented in 6^th^ decade. The majority of them have a normal healthy childhood. Age may be a risk factor for GS as arrested progenitor B cells and absent peripheral B cells are usually observed in late-onset GS. Further studies in age-dependent epigenetics in B cell lymphopoiesis may be useful in understanding this [5].

Thymoma is usually detected earlier than immunodeficiency in about half of patients [6]. Similarly in our patient immune dysfunction was evidenced by frequent infections 6 months after the diagnosis of thymoma. Type AB thymoma, spindle cell and lymphoepithelial thymoma are found to be most commonly associated with GS. In concordance with this our patient had Type AB Thymoma [3].

A study on 162 patients with GS showed that nearly 95% of the patients had low or absent B cells and a vast majority had hypogammaglobulinemia [3]. Cellular immunity is predominantly affected in GS and patients may have low CD4 count, elevated CD8 count, or altered CD4/CD8 ratio. Our patient had hypogammaglobulinemia and absent peripheral B cells, but her quantitative T cell measures were within normal limits.

Hypogammaglobulinemia is a feature shared by CVID, X-linked agammaglobulinaemia (XLA) and GS. However, the pathogenesis in these three conditions appears to be different. In CVID, isotype-switched CD27+ memory B cells are particularly reduced which may be partly due to defects in toll-like receptors 7 and 9 [7]. In XLA the defect appears to be at the pre-BI and pre-BII cell stage, whereas in GS it is probably at the earlier pro-B cell stage [5]. The association of thymoma and hypogammaglobulinemia is observed to have worse prognosis than other types of hypogammaglobulinemia because of associated defects also in the cellular immune response, resulting not only in bacterial but also viral, fungal and parasitic infections [8].

Most common sites of infections observed in GS are the sino-pulmonary tract, gastrointestinal tract and liver, skin and soft tissue and mucosa. Patients may also present with features of autoimmune dysregulation in the form of pure red cell aplasia, myasthenia gravis and lichen planus. Our patient had manifestations of autoimmune dysregulation (lichen planus), humoral immune defects (eg: Giardia), cellular immune defects (eg: CMV, Candida) and phagocytic defects (EPEC). Rarely solid and hematological malignancies and features of bone marrow dysfunction like anemia, lymphocytopenia, CD4 lymphopenia, neutropenia and eosinopenia may also be observed in patients with GS [5].

Treatment of thymoma is mainly by surgical removal or debulking of the tumor. Stage 3 or 4 thymoma may require radiotherapy and combination chemotherapy. Completeness of tumor resection is considered as the most important predictor of long-term prognosis. Treatments for GS are mainly supportive with antimicrobials and immunoglobulin replacement.

Studies have shown that thymectomy is not a useful treatment for the autoimmune disorders; however, it is superior to conservative medical therapy in uncomplicated myasthenia gravis. It is recommended in most cases of ocular and juvenile myasthenia. Thymus derived B cells and plasmablasts persist in tissues after thymectomy. In the case of Good’s syndrome, thymectomy does not provide a better prognosis; neither does it restore immunological function [8].

We have come a long way in understanding GS, but many lacunae continue to persist. From the incomplete definition of GS to the unvalidated hypothesis regarding the etiology and mechanism of disease, there is a definite need for further multicentre longitudinal studies to reach a consensus in management of GS.

## Conclusion

Good syndrome is a rare combined immunodeficiency in adults. Infection type and site impact its overall survival. Our case highlights the importance of clinical suspicion for adult-onset immune dysfunction like Good syndrome in at-risk patients who present with multiple bouts of infection, particularly in thymoma cases. A prompt diagnosis and Immunoglobulin replacement will help in clinical improvement and reduction in recurrent infections as well as hospitalizations which were observed in our patient. Clinicians should be aware that thymoma can precede the onset of immunodeficiency. It is very crucial to follow up those patients closely. We recommend prescribing an electrophoresis of proteins in all case of thymoma to search for hypogammaglobulinemia and GS.
